# Identifying the Risk Factors for Orbital Complications in Isolated Sphenoid Rhinosinusitis

**DOI:** 10.3390/medicina60010128

**Published:** 2024-01-10

**Authors:** Shiaw-Yu Chang, Chi-Che Huang, Yu-Hsi Fan, Pei-Wen Wu, Ta-Jen Lee, Po-Hung Chang, Chien-Chia Huang

**Affiliations:** 1 Division of Rhinology, Department of Otolaryngology, Chang Gung Memorial Hospital and Chang Gung University, Taoyuan 333, Taiwan; char2631@gmail.com (S.-Y.C.); hcc3110@cgmh.org.tw (C.-C.H.); indexfan@cgmh.org.tw (Y.-H.F.); a9665@cgmh.org.tw (P.-W.W.); entlee@cgmh.org.tw (T.-J.L.); bc1766@gmail.com (P.-H.C.); 2 School of Medicine, Chang Gung University, Taoyuan 333, Taiwan; 3 Department of Otolaryngology, Xiamen Chang Gung Hospital, Xiamen 361028, China

**Keywords:** bony dehiscence, computed tomography, orbital complication, rhinosinusitis, sphenoid sinus

## Abstract

*Background and Objectives*: Isolated sphenoid rhinosinusitis may have devastating consequences such as orbital complications due to its anatomical contiguity with vital structures. This study aimed to identify patients with isolated sphenoid inflammatory diseases at high risk for developing orbital complications and requiring aggressive management through investigation of the clinical and computed tomography (CT) characteristics of patients with isolated sphenoid rhinosinusitis. *Materials and Methods*: The medical records of patients who underwent endoscopic sinus surgery between 2005 and 2022 were retrospectively reviewed. Patients with isolated sphenoid rhinosinusitis were identified based on a manual review of the clinical and histopathological findings. Participants’ clinical and CT features were reviewed. *Results*: Among the 118 patients with isolated sphenoid rhinosinusitis, 15 (12.7%) developed orbital complications, including diplopia, extraocular motility limitation, ptosis, and visual impairment. Headaches and facial pain occurred significantly more frequently in patients with orbital complications than in those without orbital complications (*p* < 0.001). Patients with diabetes mellitus or malignant neoplasms were more likely to develop orbital complications than those without these comorbidities (*p* < 0.05). Bony dehiscence on CT images was significantly more common in patients with orbital complications than in those without. In the regression analysis, diabetes mellitus (OR, 4.62), malignant neoplasm (OR, 4.32), and bony dehiscence (OR, 4.87) were significant predictors of orbital complications (*p* < 0.05). *Conclusions*: Headaches and facial pain are the most common symptoms of isolated sphenoid rhinosinusitis. Orbital complications of isolated sphenoid rhinosinusitis are more common in patients with comorbidities such as diabetes mellitus or malignancy or in those with bony dehiscence on CT images.

## 1. Introduction

Rhinosinusitis is defined as inflammation of the nasal cavity and paranasal sinuses, characterized by clinical symptoms such as nasal obstruction or nasal discharge accompanied by facial pain or loss of smell [1,2]. Isolated sphenoid rhinosinusitis, in which inflammation occurs exclusively in the sphenoid sinus, is relatively rare. According to previous research, isolated sphenoid sinusitis accounts for only 2~3% of all paranasal sinus infections [3,4].

The development of isolated sphenoid rhinosinusitis is not fully understood. Bacterial rhinosinusitis and fungal ball are the most common inflammatory diseases of the sphenoid sinus [4,5,6]. Surgical intervention should be considered for bacterial rhinosinusitis refractory to conservative medical therapy and suspected fungal ball [6]. However, patients with isolated sphenoid rhinosinusitis usually experienced vague nasal symptoms and present with nonspecific symptoms such as a headache, facial pain, and fullness [3,4,7,8]. On the other hand, appropriate imaging techniques, for example, computed tomography, are essential for diagnosis [6,9]. Therefore, isolated sphenoid rhinosinusitis is difficult to diagnose and is easily neglected.

Furthermore, isolated sphenoid rhinosinusitis may have devastating consequences such as orbital complications due to its anatomical contiguity with vital structures including the cranial nerves and cavernous sinus [10,11,12,13,14]. For the orbital complications secondary to isolated sphenoid rhinosinusitis, previous case reports have demonstrated cavernous sinus thrombophlebitis and optic neuritis [10,11,12,13,14]. The sixth cranial nerve (abducens nerve) and optic nerve were consequently affected, which resulted in ophthalmologic symptoms including diplopia, extraocular motility limitation, orbital pain, and visual impairment. In two of the cases mentioned above, the orbital symptoms improved after receiving intravenous antibiotic treatment and prompt endoscopic sinus surgery [13]. However, visual impairment deterioration was found in the other case due to delayed surgical drainage [14]. Thus, the early identification of cases with isolated sphenoid rhinosinusitis, the identification of associated risk factors for developing orbital complications, and establishing appropriate therapeutic modalities are important tasks.

Isolated sphenoid rhinosinusitis can be generally classified as fungal rhinosinusitis and non-fungal rhinosinusitis based on the histopathologic evidence [6]. Among the subgroup of fungal rhinosinusitis, isolated sphenoid invasive fungal rhinosinusitis has been assumed to cause neuro-ophthalmologic disorder by invading the bony wall of the sphenoid sinus and surrounding vital structures [14,15]. Apart from invasive fungal rhinosinusitis, other types of isolated sphenoid rhinosinusitis could also result in ophthalmologic symptoms. Although the detailed mechanism remains unclear, orbital complications including diplopia, extraocular motility limitation, ptosis, and visual impairment may be caused by either inflammation involving the optic nerve, orbital apex, or cavernous sinuses, or a mass effect in the case of fungal balls [14]. To prevent profound sequela, medical intervention should be performed in a timely manner.

Therefore, this study aimed to identify patients with isolated sphenoid inflammatory diseases at high risk of developing orbital complications and requiring aggressive management through investigation of the clinical and computed tomography (CT) characteristics of patients with isolated sphenoid rhinosinusitis.

## 2. Materials and Methods

**Figure 1:**
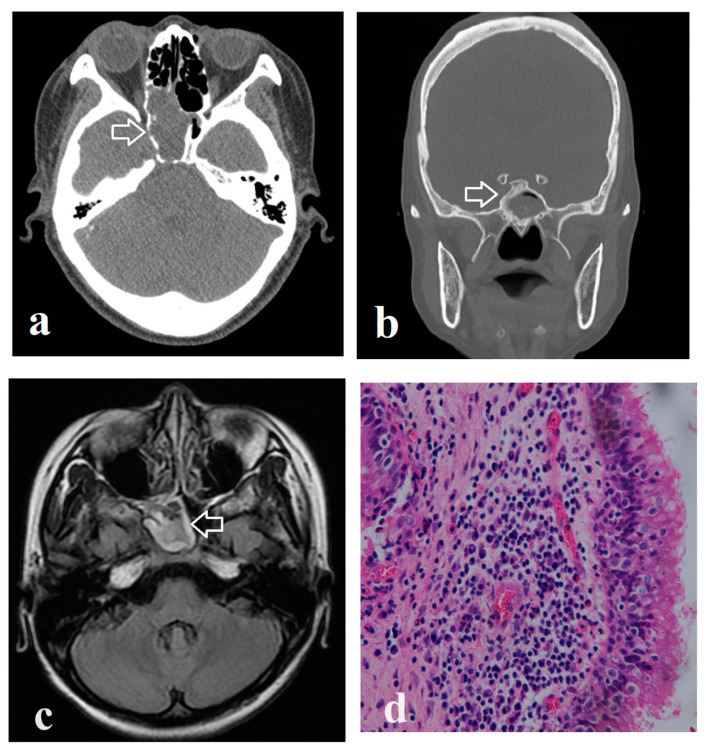
Computed tomographic features, magnetic resonance (MR) image, and histopathology of isolated sphenoid rhinosinusitis. (**a**) Total opacification and bony dehiscence (arrow); (**b**) partial opacification and lateral wall sclerosis (arrow); (**c**) right isolated sinusitis on MR image (arrow); (**d**) inflammation without fungus invasion in sinonasal mucosa, Magnification: 400×.

The medical records of patients who underwent endoscopic sinus surgery between 2005 and 2022 were retrospectively reviewed. Patients histopathologically diagnosed with invasive fungal sinusitis (evidence of fungus penetrating the host tissue), sinonasal tumor, mucocele, or pathologies other than inflammation were excluded. A total of 118 patients with isolated sphenoid rhinosinusitis were identified based on a manual review of the clinical and histopathological findings. This could include a unilateral or bilateral lesion. All patients underwent endoscopic sphenoidotomy. The affected sinus was widely opened and the intrasinus fungal ball or inspissated mucopurulent material was meticulously removed. The affected sinus was irrigated with normal saline according to the surgeon’s preference. Participants’ demographic data including age and sex, clinical symptoms, underlying comorbidities, and laboratory test results were retrieved from their medical records. In addition, CT images for each patient were also collected and interpreted. The following features were determined using CT: the site of the sphenoid-sinus lesion, total or partial opacification, intrasinus lesion surface irregularities, bony dehiscence, lateral-wall sclerosis, and intralesional hyperdensity (Figure 1a,b). The above features were defined according to a previous study [6]. The Institutional Review Board approved this study (approval number: 202201253B0) on 25 August 2022. The requirement for informed consent was waived because of the retrospective nature of the study and the anonymity of the data. The STROBE reporting guidelines were used in manuscript preparation.

Statistical analyses were performed using SPSS Statistics v27.0. (IBM Corp, Armonk, NY, USA), and the data are presented as mean ± standard deviation. Fisher’s exact test (for categorical variables) and the Mann–Whitney U test (for continuous variables) were used for comparisons between participants with and without orbital complications. Univariate and multivariate logistic regression analyses were performed to examine the associations between orbital complications and variables by calculating the odds ratios (ORs) with 95% confidence intervals. Statistical significance was set at *p* < 0.05.

## 3. Results

Among the 118 patients with isolated sphenoid rhinosinusitis, 15 (12.7%) developed orbital complications, including diplopia, extraocular motility limitation, ptosis, and visual impairment. Orbital complications such as optic neuritis, ocular hypertension, and oculomotor nerve palsy were observed in five, four, and three patients, respectively. The cavernous sinus was involved in one patient who experienced ptosis, blindness, and extraocular motility limitation. Another patient diagnosed with orbital apex syndrome complained of the same symptoms as above. All patients with orbital complications received parenteral antibiotics including amoxycillin + clavulanic acid, ceftriaxone, cefazolin, and clarithromycin from the time of arrival in the emergency room. Amphotericin B and voriconazole were used as the initial drugs in three and two patients, respectively; however, they were discontinued within two weeks in all patients after invasive fungal infection was histopathologically excluded (Figure 1d). The characteristics of participants with and without orbital complications are summarized in Table 1. The mean age of both groups was in the mid-fifties and there was no significant difference between the groups. There were four pediatric patients, including one patient in the subgroup of orbital complications. Sixty-nine patients were histopathologically diagnosed with fungal balls and forty-nine patients had non-fungal rhinosinusitis. A female predominance was noted in both groups, and there was no significant sex difference between the two groups. Headaches and facial pain occurred significantly more frequently in patients with orbital complications than in those without orbital complications (*p* < 0.001). Patients with diabetes mellitus or malignant neoplasms were more likely to develop orbital complications than those without these comorbidities (*p* < 0.05). Among the twelve patients who had a history of malignancy, two patients had cancer in the head and neck region (nasopharyngeal cancer and external ear cancer), and two and eight patients suffered from hematologic malignancy and other solid tumors, respectively. Eleven of these patients had received chemotherapy for their malignancy. Regarding the CT features, rhinosinusitis was limited to the unilateral or bilateral sphenoid sinuses. Further, bony dehiscence was significantly more common in patients with orbital complications than in those without. Ten patients, including two with orbital complications, had a previous history of endoscopic sinus surgery, and three of them had nasal polyps. There were no differences between the groups.

The results of the logistic regression analysis are presented in Table 2. In the univariate analysis, diabetes mellitus (OR, 4.62), malignant neoplasms (OR, 4.32), and bony dehiscence (OR, 4.87) were significant predictors of orbital complications (*p* < 0.05). These three variables remained significant predictors of orbital complications in the multivariate analysis.

## 4. Discussion

Concerning the patient selection in this retrospective cohort, we should emphasize that patients diagnosed with invasive fungal sinusitis, sinonasal tumors, mucocele, or pathologies other than inflammation were already excluded. The diagnosis of all included participants was based on histopathological examination post-operatively. The reason for this was that we deliberately focused on the orbital complications resulting from inflammation in isolated sphenoid rhinosinusitis patients, which has not been comprehensively investigated. In contrast, invasive fungal rhinosinusitis, for example, is a known lethal disease with high mortality and morbidities, and orbital involvement is one of the critical complications [15,16]. The mechanism of ophthalmologic disorder was considered as the rapid invasion of blood vessels by hyphae, inducing luminal thrombosis and finally causing tissue necrosis [17]. In addition, bacterial rhinosinusitis and fungal balls are more common for sphenoid inflammatory diseases in clinical practice compared to invasive fungal rhinosinusitis [3].

Regarding the orbital complications secondary to isolated sphenoid rhinosinusitis, previous case reports have demonstrated cavernous sinus thrombophlebitis and optic neuritis [10,11,12,13,14,18,19]. The sixth cranial nerve (abducens nerve) and optic nerve were consequently affected, which resulted in ophthalmologic symptoms including diplopia, extraocular motility limitation, orbital pain, and visual impairment. In two of the cases mentioned above, the orbital symptoms improved after receiving intravenous antibiotic treatment and prompt endoscopic sinus surgery [13,18]. However, visual impairment and deterioration were observed in the other case due to delayed surgical drainage [19]. As for our subjects in this manuscript, the ophthalmologic complaints that were documented included blurred vision, diplopia, extraocular motility limitation, ptosis, and eye pain. Except for the two patients diagnosed with cavernous sinus thrombosis and orbital apex syndrome, respectively, all other patients achieved favorable clinical outcomes (partial or even total recovery in visual function) after surgery.

In this study, headaches and facial pain were the most common isolated sphenoid rhinosinusitis symptoms, particularly in patients with orbital complications (93.3%). This finding is in line with previous studies [5]. Charakorn et al. reported that headaches are the most common clinical manifestation [8]. In patients with isolated sphenoid rhinosinusitis, we speculated that headaches or facial pain may increase the possibility of impending orbital or intracranial complications. Headache characteristics, including their location, were not available in more than half of our cohort with orbital complications due to the retrospective nature of the study. However, a previous study that investigated the headache characteristics in patients with isolated sphenoid sinus disease showed that the locations of headaches varied widely, including the vertex (24%) and hemicranial (19%), retroorbital (14%), diffuse (14%), bifrontal (14%), occipital (10%), and fronto-orbital (5%) regions [20]. The mechanism of these headaches is considered to be irritation of the trigeminal nerve via the sphenopalatine or nasociliary nerves, which innervate the sphenoid sinus [20].

Nasal symptoms, including nasal obstruction, post-nasal dripping, and rhinorrhea, were not as common in patients with isolated sphenoid rhinosinusitis as in those with other types of rhinosinusitis [1,2]. Nasal symptoms were extremely rare in patients with orbital complications in this study because of the focus on orbital evaluation during management. Another consideration is that patients may turn to our medical center if there is a relative emergent condition such as orbital complications.

The female predominance (66.9%) in isolated sphenoid rhinosinusitis may be due to the inclusion of patients with fungal balls. Fungal balls are more common in females than in males and in those in their fifties [6,21,22]. Although a consensus on the explanation for this phenomenon is lacking, the longer life expectancy and the effect of menopause in females have been proposed as possible causes [21,23]. Our previous study observed that the female-to-male ratio was highest at 51–60 years of age (2.02). The mean age (SD) at menopause in Taiwan is 50.2 (±4.0) years [24]. This suggests that post-menopausal hormonal changes may be associated with the formation of sinonasal fungus balls. Although the precise mechanism is not clear, the nasal mucosa is affected by changes in female sex hormones [25]. Ozler et al. and Gumussoy et al. both reported on the prolongation of nasal mucociliary clearance times in menopausal women [26,27]. Impaired mucociliary activity may weaken the defense mechanism of the nasal respiratory epithelium, resulting in the failed clearance of airborne fungal spores and the formation of sinonasal fungus balls.

In addition, the etiologies also reflect the age of isolated sphenoid rhinosinusitis. Sphenoid fungal balls are less commonly observed in the pediatric population than in patients with an older age [6]. According to a previous article, most pediatric patients with sphenoid rhinosinusitis are adolescents or pre-adolescents [28,29]. Bacterial rhinosinusitis has been shown to be the predominant etiology, with the pathogens including Staphylococcus aureus and various Streptococcus species [28]. In our database, no pediatric patient was noted in the subgroup of fungal rhinosinusitis. Interestingly, only one patient (16-year-old) in the pediatric population suffered from orbital complications. However, the patient was also comorbid with a hematologic malignancy (acute lymphocytic leukemia). Therefore, we deduce that age may be related to the histopathological etiology instead of the incidence of orbital complications in isolated sphenoid rhinosinusitis.

In this study, patients with isolated sphenoid rhinosinusitis and diabetes mellitus or malignant neoplasms were prone to orbital complications. A previous study reported that high blood glucose and C-reactive protein levels were risk factors for orbital complications in patients with non-invasive fungal rhinosinusitis [30]. Those laboratory data suggested an immunosuppressive status and systemic inflammation [30]. Patients with diabetes mellitus or malignant neoplasms are usually considered relatively immunocompromised and could be susceptible to complications of rhinosinusitis [31,32]. In a review article by Nyunt et al., they reported that the immune responses of innate and adaptive immune systems were defective [33]. A hyperglycemic state altered the structure of complement and activated protein kinase C, which inhibited immune cell activity. A malignant neoplasm is one of the most common causes of an immunocompromised status. Cancer cells produce a variety of immunosuppressive factors which consequently create an immunosuppressive microenvironment [34,35]. In our study, two patients had cancer in the head and neck region (nasopharyngeal cancer and external ear cancer). Two and eight patients suffered from a hematologic malignancy and other solid tumors, respectively. Eleven of patients with a previous history of cancer had received chemotherapy for their malignancy. Chemotherapy is also a well-known etiology of an immunocompromised status [36].

CT is the most frequently used modality for evaluating rhinosinusitis, and is particularly helpful in evaluating bone alignment [37]. A previous study revealed that chronic inflammation in the sinus may affect the underlying bone and contribute to different degrees of bony dehiscence [38]. Bone remodeling within the sinonasal cavity occurs in the inflammatory process and is influenced by many factors at the cellular level [39]. For example, transforming growth factor (TGF)-β can upregulate osteoblast and osteoclast activity, which was observed in a mouse model [40]. Moreover, bone thickening was thought to be important evidence to evaluate osteitis in patients with chronic rhinosinusitis [41,42]. Based on our previous study, lateral wall sclerosis and bony dehiscence are more common in patients with sphenoid sinus fungal balls than unilateral sphenoid rhinosinusitis [6]. Nevertheless, the relationship between these two CT image features is not fully understood. In our statistical analysis, there is no significant difference between patients with and without orbital complications for the presence of lateral wall sclerosis. Although these two radiographic characteristics may both indicate potential inflammatory reactions within the underlying bone, we suggest that only bony dehiscence plays a role in the process of developing orbital complications. Our study demonstrates that patients with isolated sphenoid rhinosinusitis with bony dehiscence on CT are at risk for orbital complications (OR, 4.87) and might require more aggressive treatment. CT is usually performed as the first imaging study in the diagnostic workup of patients with sinonasal disorders. However, magnetic resonance imaging (MRI), which has superior soft-tissue resolution, is required in patients with suspected sphenoid-sinus disease with intracranial or intraorbital invasion, neoplasms, and sphenoid sinus-wall erosion or any uncertainty on CT [5,6,7]. This study identified patients at high risk of developing orbital complications among those with isolated sphenoid inflammatory diseases. These patients may require further MRI evaluation (Figure 1c).

The sphenoid sinus is located in the center of the skull. Many vulnerable structures surround this sinus, for example, the dura mater, cranial nerves (III, IV, V1, V2, and VI), the optic nerve and chiasm, the internal carotid artery, the cavernous sinus, the pituitary gland, the sphenopalatine ganglion, the sphenopalatine artery, and the pterygoid canal [4,43]. The associated symptoms may refer to these structures rather than involving the sinus. Because of its deep-seated anatomy, this sinus does not usually present with nasal symptoms such as nasal obstruction or rhinorrhea [4,5,6]. The most common symptom is headache, which has a prevalence ranging from 28% in tumor lesions to 98% in inflammatory lesions [44]. The next most common symptoms are cranial nerve deficit, visual alteration such as visual loss or diplopia, and pain or numbness according to trigeminal nerve involvement. These indicate a possible orbital or intracranial complication requiring prompt appropriate management such as surgical intervention. Nowadays, CT and MR imaging are used to evaluate patients with suspected neurological problems; incidental abnormalities of the sphenoid sinus are noted for further management. However, CT is the first-line imaging modality for evaluating paranasal sinus diseases because it provides high-resolution images of the anatomic structure surrounding the paranasal sinuses and the areas affected by the lesion [33,45]. Previous studies have explored the CT features of patients with isolated sphenoid sinus fungus ball (SSFB) [6]. Several CT imaging features, including surface irregularity, inner sinus wall erosion, lateral sinus wall sclerosis, and intralesional hyperdensity, have been proposed to predict SSFB. Headaches, rhinorrhoea, nasal obstruction, postnasal dripping, and hyposmia were the most common symptoms. In the univariate analysis, older age, lower white blood cell counts, irregular surface, bony dehiscence, lateral wall sclerosis, and intralesional hyperdensity were significant predictors for SSFB. Older age, irregular surface, and intralesional hyperdensity remained statistically significant in the multivariate analysis. Based on the results of the regression analysis, a nomogram for predicting the probability of SSFB was proposed. Therefore, in this study, we aimed to identify patients with isolated sphenoid inflammatory diseases at high risk of developing orbital complications and requiring aggressive management through investigation of the clinical and CT characteristics of patients with isolated sphenoid rhinosinusitis. All patients in our study cohort received a fine-section CT image examination before surgery. Based on the CT findings and clinical characteristics, this study seeks to help clinicians to predict the risk of developing orbital complications in each patient and to plan appropriate treatment modalities for patients. Our results show that comorbid diabetes mellitus (OR, 4.62), malignant neoplasms (OR, 4.32), and bony dehiscence on CT images (OR, 4.87) are significant predictors of orbital complications. Aggressive management should be implemented as soon as possible for these patients.

This study had some limitations. First, patients with isolated sphenoid rhinosinusitis who did not undergo surgery were excluded. This may have led to selection bias. The prevalence of orbital complications may be overestimated. However, as isolated sphenoid rhinosinusitis may have devastating consequences such as orbital complications due to its anatomical contiguity with vital structures including the cranial nerves and cavernous sinus, identifying risk factors for developing orbital complications is significantly important. Second, clinicians need to consider differential diagnoses beyond isolated sphenoid rhinosinusitis, such as neoplasms, mucoceles, encephaloceles, and vascular lesions. During daily clinical practice, nasal endoscopic examination and associated findings including the absence of purulent nasal secretions or the presence of mass lesions are usually helpful initially. Nevertheless, bone erosion or destruction is a common CT finding in these pathologies, and further evaluation using magnetic resonance imaging or endoscopic biopsy is necessary. In this study, we investigated the clinical and CT characteristics of isolated sphenoid rhinosinusitis and identified the risk factors for orbital complications. Third, detailed clinical information such as the headache characteristics, the use of MRI, and the results of fungal examination was lacking in most cases due to the retrospective nature of the study and the rarity of the clinical condition. Last but not least, the patients included in this study all attended one single tertiary medical center. Future prospective studies with a comprehensive evaluation of the clinical and imaging information are necessary to validate our results. Our study aimed to identify patients at high risk for developing orbital complications and requiring aggressive treatment. However, ideal treatment strategies such as the usage of anti-fungal agents, the usage of corticosteroids, and the timing of surgery may be investigated going forward.

## 5. Conclusions

Headaches and facial pain are the most common symptoms of isolated sphenoid rhinosinusitis. Orbital complications of isolated sphenoid rhinosinusitis are more common in patients with comorbidities such as diabetes mellitus or malignancies or in those with bony dehiscence on CT images.

## Figures and Tables

**Table 1:** Characteristics and computed tomographic features of the participants.

Variables	Orbital Complication	No Orbital Complication	*p* Value
	(n = 15)	(n = 103)	
Age, years (mean ± SD)	58.9 ± 21.1	52.6 ± 16.1	0.116
Gender			
Male, n	6 (40.0%)	33 (32.0%)	0.565
Female, n	9 (60.0%)	70 (68.0%)	
Site of sphenoid lesion			
Left, n	5 (33.3%)	47 (45.6%)	0.149
Right, n	8 (53.3%)	53 (51.5%)	
Single sphenoid sinus, n	2 (13.3%)	3 (2.9%)	
Histopathology			
Fungus ball, n	7 (46.7%)	62 (60.2%)	0.403
Non-fungus, n	8 (53.3%)	41 (39.8%)	
Laboratory data			
WBC count, k per µl	7.52 ± 1.79	6.83 ± 2.15	0.050
Underlying conditions			
Diabetes mellitus, n	6 (40.0%)	13 (12.6%)	0.016 *
Malignant neoplasms, n	4 (26.7%)	8 (7.8%)	0.046 *
Previous sphenoid sinus surgery, n	2 (13.3%)	19 (18.4%)	1.000
Clinical presentations			
Headache and facial pain, n	14 (93.3%)	47 (45.6%)	<0.001 ***
Rhinorrhea, n	3 (20.0%)	49 (47.6%)	0.054
Purulent rhinorrhea, n	3 (20.0%)	25 (24.3%)	1.000
Bloody rhinorrhea, n	0 (0%)	11 (10.7%)	0.355
Nasal obstruction, n	0 (0%)	45 (43.7%)	<0.001 ***
Post nasal dripping, n	2 (13.3%)	40 (38.8%)	0.081
Hyposmia, n	2 (13.3%)	15 (14.6%)	1.000
Foul odor smell, n	0 (0%)	9 (8.7%)	0.601
Tinnitus, n	0 (0%)	2 (1.9%)	1.000
Incidental found, n	0 (0%)	8 (7.8%)	0.594
Features of CT image			
Total opacification, n	7 (46.7%)	49 (47.6%)	1.000
Partial opacification, n	8 (53.3%)	54 (52.4%)	1.000
Irregular surface, n	3 (20.0%)	34 (33.0%)	0.385
Bony dehiscence, n	10 (66.7%)	30 (29.1%)	0.007 **
Lateral wall sclerosis, n	9 (60.0%)	58 (56.3%)	1.000
Intralesional hyperdensity, n	4 (26.7%)	38 (36.9%)	0.569

CT, computed tomography; SD, standard deviation; WBC, white blood cell. * *p* < 0.05, ** *p* < 0.01, *** *p* < 0.001.

**Table 2:** Logistic regression analyses of the associated factors of orbital complications.

	Univariate Analysis		Multivariate Analysis	
Variables	Odds Ratio (95% CI)	*p* Value	Odds Ratio (95% CI)	*p* Value
Characteristics of patients				
Age	1.02 (0.99–1.06)	0.179	1.00 (0.96–1.04)	0.892
Diabetes mellitus	4.62 (1.41–15.10)	0.011 *	5.27 (1.40–19.93)	0.014 *
Malignant neoplasms	4.32 (1.12–16.71)	0.034 *	5.23 (1.17–23.31)	0.030 *
Female sex	0.71 (0.23–2.15)	0.542		
WBC (x1000 per μL)	1.14 (0.91–1.43)	0.243		
CT imaging features				
Total opacification	0.96(0.33–2.86)	0.948		
Partial opacification	1.04 (0.35–3.07)	0.948		
Irregular surface	0.51 (0.13–1.92)	0.317		
Bony dehiscence	4.87 (1.53–15.44)	0.007 **	5.21 (1.44–18.84)	0.012 *
Lateral wall sclerosis	1.16 (0.39–3.51)	0.788		
Intralesional hyperdensity	0.62 (0.19–2.09)	0.443		

CI, confidence interval; WBC, white blood cell; CT, computed tomography. * *p* < 0.05, ** *p* < 0.01.

## Data Availability

All data described in this study are presented in the manuscript. The datasets analyzed are available from the corresponding author upon reasonable request.
